# New *Trypanosoma evansi* Type B Isolates from Ethiopian Dromedary Camels

**DOI:** 10.1371/journal.pntd.0004556

**Published:** 2016-04-01

**Authors:** Hadush Birhanu, Tadesse Gebrehiwot, Bruno Maria Goddeeris, Philippe Büscher, Nick Van Reet

**Affiliations:** 1 College of Veterinary Medicine, Mekelle University, Mekelle, Ethiopia; 2 KU Leuven, Faculty of Bioscience Engineering, Department of Biosystems, Leuven, Belgium; 3 Institute of Tropical Medicine, Department of Biomedical Sciences, Antwerp, Belgium; RTI International, UNITED STATES

## Abstract

**Background:**

*Trypanosoma (T*.*) evansi* is a dyskinetoplastic variant of *T*. *brucei* that has gained the ability to be transmitted by all sorts of biting flies. *T*. *evansi* can be divided into type A, which is the most abundant and found in Africa, Asia and Latin America and type B, which has so far been isolated only from Kenyan dromedary camels. This study aimed at the isolation and the genetic and phenotypic characterisation of type A and B *T*. *evansi* stocks from camels in Northern Ethiopia.

**Methodology/principal findings:**

*T*. *evansi* was isolated in mice by inoculation with the cryopreserved buffy coat of parasitologically confirmed animals. Fourteen stocks were thus isolated and subject to genotyping with PCRs targeting type-specific variant surface glycoprotein genes, mitochondrial minicircles and maxicircles, minisatellite markers and the F1-ATP synthase γ subunit gene. Nine stocks corresponded to type A, two stocks were type B and three stocks represented mixed infections between A and B, but not hybrids. One *T*. *evansi* type A stock was completely akinetoplastic. Five stocks were adapted to *in vitro* culture and subjected to a drug sensitivity assay with melarsomine dihydrochloride, diminazene diaceturate, isometamidium chloride and suramin. *In vitro* adaptation induced some loss of kinetoplasts within 60 days. No correlation between drug sensitivity and absence of the kinetoplast was observed. Sequencing the full coding sequence of the F1-ATP synthase γ subunit revealed new type-specific single nucleotide polymorphisms and deletions.

**Conclusions/significance:**

This study addresses some limitations of current molecular markers for *T*. *evansi* genotyping. Polymorphism within the F1-ATP synthase γ subunit gene may provide new markers to identify the *T*. *evansi* type that do not rely on variant surface glycoprotein genes or kinetoplast DNA.

## Author Summary

*Trypanosoma (T*.*) evansi* causes surra in various animal species in Africa, Latin America and Asia. Despite inducing important animal suffering, economic losses and being a World Animal Health Organisation (OIE) notifiable disease, surra is severely neglected in terms of awareness, control interventions and research into improved control tools. Most serological tests can only detect *T*. *evansi* type A, while molecular tests rely on detection of highly variable genes or on fragile kinetoplast DNA. Even more, the obscure *T*. *evansi* type B, first isolated decades ago in Kenya, totally escapes surveillance due to absence of reliable diagnostic tools. In the present study we isolated new type B stocks from Ethiopia, thus suggesting that this type of *T*. *evansi* is probably more widely distributed than previously thought. We further report on an alternative molecular marker for both types of *T*. *evansi* and present data on the drug sensitivity of the Ethiopian isolates.

## Introduction

Surra, a wasting disease caused by *Trypanosoma (T*.*) evansi*, is one of the non tsetse-transmitted Animal African Trypanosomoses (AAT) occurring in Ethiopia. The disease imposes significant financial losses due to reduced fertility and mortality and is prohibiting the import of highly productive yet trypanosusceptible cattle breeds [1–3]. *T*. *evansi* belongs to the subgenus *Trypanozoon*, that also comprises *T*. *brucei* and *T*. *equiperdum* [4–6]. In terms of geographical distribution, *Trypanosoma equiperdum* and *T*. *evansi*, causing respectively dourine in horses and surra in livestock in Africa, Asia, and South America, have been far more successful than *T*. *brucei*, a parasite confined to sub-Saharan Africa where its vector, the tsetse fly, is present [7]. Recent phylogenetic studies suggest that *T*. *evansi* and *T*. *equiperdum* evolved from *T*. *brucei* on several occasions and from genetically distinct *T*. *brucei* strains and therefore could be considered as subspecies of *T*. *brucei* [8,9].

Trypanosomes are characterised by the presence of a structure called kinetoplast that corresponds with the DNA (kDNA) of their unique mitochondrion. *T*. *brucei* kDNA contains 20–50 copies of maxicircles (about 23 kb) and a highly diverse set of thousands of minicircles (about 1 kb). Maxicircles contain rRNA coding regions and genes coding for subunits of the respiratory chain complexes while minicircles code for guide RNAs required for editing [10].

*T*. *equiperdum* and *T*. *evansi* are dyskinetoplastic (kDNA-) since they lack part of the kDNA [8–11]. *T*. *equiperdum* typically has retained maxicircles, in some cases with substantial deletions, but has lost its minicircle diversity. *T*. *evansi* does not have maxicircles and either shows minicircle homogeneity or are akinetoplastic (kDNA) [10,12–14]. Based on their minicircle restriction digestion profile, *T*. *evansi* can be divided into type A and type B [15,16].

*T*. *evansi* type A is the most abundant and is found in Africa, South America and Asia. It is characterised by the presence of the gene for the variant surface glycoprotein (VSG) RoTat 1.2. This RoTat 1.2 VSG is expressed early during infections resulting in the detectability of anti-RoTat 1.2 antibodies in animals infected with *T*. *evansi* type A [17,18]. In contrast, *T*. *evansi* type B is far less common and has so far been isolated only from camels in Kenya [16,19]. More recently, serological and molecular evidence for the presence of *T*. *evansi* type B in Sudan, Ethiopia and Chad was published [20–24]. *T*. *evansi* type B lacks the RoTat 1.2 gene and as a consequence, infections with this type are not detected with serological and molecular tests based on RoTat 1.2 VSG, such as the CATT/*T*. *evansi* and RoTat 1.2 PCR [15,18,19,25]. So far, three molecular tests have been developed for the identification of *T*. *evansi* type B: the EVAB PCR, targeting a type B-specific minicircle DNA sequence, and a PCR and a LAMP targeting a type B-specific VSG JN 2118Hu [15,19,26]. *T*. *equiperdum* is the least known parasite of the *Trypanozoon* group, with very few isolates available for research, albeit new stocks were isolated from Ethiopian and Venezuelan horses recently [24,27].

Unlike *T*. *brucei*, *T*. *evansi* and *T*. *equiperdum* cannot develop in tsetse flies due to their inability to transform into the procyclic life stage. They can only survive in a mammalian host where they produce ATP exclusively through glycolysis. In contrast to bloodstream forms, ATP production in procyclic trypanosomes relies on oxidative phosphorylation and, therefore, on the capacity to express the full set of corresponding mitochondrial genes, including some which are encoded by the kDNA [10,28]. Bloodstream forms of *T*. *evansi*, *T*. *equiperdum* and laboratory-generated *T*. *brucei* strains that have lost all or critical parts of their kDNA, can survive without kDNA due to specific single amino acid mutations in the gamma (γ) subunit of the mitochondrial F1-ATP synthase [28]. Interestingly, the specific mutations/deletions in the C-terminal region of F1-ATP synthase γ subunit enable differentiation among the *Trypanozoon* strains [8]. Furthermore, when the F1-ATP synthase γ subunits of *T*. *evansi* type A (A281del), *T*. *equiperdum* (A273P) and the laboratory-generated *T*. *brucei* (L262P) strains are overexpressed in a *T*. *brucei* γ subunit knock out strain, the latter can survive after loss of its kinetoplast after treatment with DNA intercalating drugs such as acriflavin or ethidium bromide [28,29]. Once the genetically modified *T*. *brucei* are independent from kDNA maintenance and expression, they become multidrug resistant to the diamidine and phenanthridine class of drugs [30].

In *T*. *evansi*, drug resistance has been reported in several type A strains originating from Africa, Asia and Latin America [31–34]. Some Chinese strains appear to be innately resistant to the phenanthridine class of drugs [35]. In contrast, nothing is known on the drug susceptibly of the *T*. *evansi* type B strains. In a previous study, we reported that *T*. *evansi* infections are very common in camels, equines, cattle and small ruminants in Tigray and Afar provinces in Northern Ethiopia [20]. We also provided molecular and serological evidence that both *T*. *evansi* type A and type B occur in these provinces. In that study, of those dromedary camels that were parasitologically positive, buffy coat samples were collected and cryopreserved in liquid nitrogen for later isolation of the parasite. We here report on the isolation, adaptation to *in vitro* culture, genetic and phenotypic characterisation and *in vitro* drug sensitivity of *T*. *evansi* type A and B from Northern Ethiopia.

## Materials and Methods

### Ethics statement

The Animal Experimentation Ethics Committee (AEEC) of the Institute of Tropical Medicine (ITM) advised on the protocol for collection of blood samples from dromedary camels (EXT2012-1) and for the isolation of trypanosomes via inoculation of mice (EXT2012-2) at the College of Veterinary Medicine, Mekelle University. The study protocol for *in vivo* expansion of trypanosomes at ITM was approved by the AEEC (BM2013-1). Collecting blood from camels and experiments on mice were conducted according to the national guidelines of the Ethiopian Ministry of Livestock and Fishery Development and the Institutional Review Board of the Ministry of Science and Technology.

### *In vivo* isolation of parasites from cryopreserved buffy coat in mice

Details on the collection and cryopreservation of buffy coat samples from dromedary camels that were parasitologically confirmed in the micro haematocrit centrifugation technique have been fully described elsewhere [20]. Two hundred μl of thawed buffy coat were inoculated intraperitoneally (IP) in two 25–30 g Swiss albino mice that were immunosuppressed with 0.16 μg kg^-1^ body weight dexamethasone (Shanghai Central Pharmaceutical, China) one day prior to inoculation [36]. Parasitaemia was checked in 5 μl of tail blood using the matching method [37], starting from day 7 post-infection and subsequently on every third day. As soon as trypanosomes were detected in at least one mouse, the animal was anaesthetised (the other kept as a backup), its blood was collected on heparin by heart puncture, diluted in an equal volume of phosphate buffered saline glucose (PSG; 7.5 g/l Na_2_HPO_4_2H_2_O, 0.34 g/l NaH_2_ PO_4_H_2_O, 2.12 g/l NaCl, 10 g/l D-glucose, pH 8) and subinoculated into four naïve mice (200 μl each) which were monitored for parasitaemia as described above. Mice used as backup were euthanised when the newly infected mice became positive. When parasitemia reached about ± 10^7.8^ cells ml^−1^ of blood, two of these parasitaemic mice were euthanised (the other two were kept as back up) and blood was taken for subinoculation into four other naïve mice. This protocol was repeated until the parasitaemia reached about 10^8.4^ cells ml^−1^. At this stage the stock was considered *in vivo* adapted. All four mice were anaesthetised and exsanguinated by heart puncture in an equal volume of Triladyl-egg yolk-phosphate buffered saline glucose (TEP) cryomedium [38] for cryopreservation in 1 ml aliquots.

### *In vivo* expansion and purification of parasite populations

Cryostabilates were thawed in a water bath at 37°C and diluted in PSG to 1 trypanosome per field (± 10^5.7^ cells ml^−1^). Two-hundred μl volumes were injected IP in two naïve 20–30 g female OF-1 mice (Charles River, Belgium). Starting from three days post infection (DPI), parasitaemia was monitored daily and harvested at first peak parasitemia, typically at day 4 to 5 post-infection, as described above. Volumes of 0.5 ml of the blood were run over a mini Anion Exchange Centrifugation Technique (mAECT) column to separate the trypanosomes from the blood [39]. The trypanosomes eluted from the column were washed twice with 5 ml ice-cold PSG by centrifugation at 1500 g for 15 min. After the last centrifugation, the supernatant PSG was discarded and the trypanosome sediment was re-suspended in 100 μl of PSG. Part of this suspension was used for *in vitro* culture adaptation. The remainder was centrifuged at 1500 g for 5 min and the sediment was frozen at -80°C until DNA extraction. The isolates used for *in vivo* isolation and expansion and the corresponding *T*. *evansi* type A and B specific PCR result on their corresponding buffy coat DNA are indicated in Table 1.

**Table 1:** List of Ethiopian *T*. *evansi* isolates with data on origin and results in RoTat 1.2 PCR and EVAB PCR performed on DNA extracted from the buffy coat specimens from the infected camels. pos: positive, neg: negative.

Stabilate code	Region	District	Station	RoTat 1.2 PCR	EVAB PCR	*In vivo* subpassages before first cryostabilate	*In vivo* expansion at ITM
MCAM/ET/2013/MU/01	Afar	Megalle	Adahara	pos	neg	3	yes
MCAM/ET/2013/MU/02	Tigray	Raya-Azebo	Chercher	pos	neg	5	yes
MCAM/ET/2013/MU/03	Tigray	Raya-Azebo	Kukufto	pos	neg	5	no
MCAM/ET/2013/MU/04	Tigray	Raya-Azebo	Chercher	pos	neg	3	yes
MCAM/ET/2013/MU/05	Tigray	Raya-Azebo	Balla	pos	neg	4	yes
MCAM/ET/2013/MU/06	Tigray	Raya-Azebo	Balla	pos	neg	3	yes
MCAM/ET/2013/MU/07	Afar	Yallo	Gubidera	pos	neg	2	yes
MCAM/ET/2013/MU/08	Afar	Golina	Ullel-ella	pos	neg	2	yes
MCAM/ET/2013/MU/09	Tigray	Raya-Azebo	Kukufto	pos	neg	3	yes
MCAM/ET/2013/MU/10	Afar	Awash Fentale	Alibete	neg	pos	2	yes
MCAM/ET/2013/MU/11	Afar	Megalle	Adahara	pos	neg	3	yes
MCAM/ET/2013/MU/12	Afar	Yallo	Gubidera	pos	neg	3	no
MCAM/ET/2013/MU/13	Afar	Golina	Ullel-ella	pos	neg	3	yes
MCAM/ET/2013/MU/14	Afar	Awash Fentale	Alibete	neg	pos	3	yes
MCAM/ET/2013/MU/15	Afar	Awash Fentale	Dihoon	pos	neg	2	yes
MCAM/ET/2013/MU/16	Afar	Golina	Ullel-ella	pos	neg	2	no
MCAM/ET/2013/MU/17	Afar	Awash Fentale	Dihoon	pos	neg	2	yes
MCAM/ET/2013/MU/18	Afar	Megalle	Adahara	pos	neg	2	no
MCAM/ET/2013/MU/19	Afar	Megalle	Adahara	pos	neg	3	no
MCAM/ET/2013/MU/20	Afar	Golina	Ullel-ella	pos	neg	2	no
MCAM/ET/2013/MU/21	Afar	Megalle	Adahara	pos	neg	3	no
MCAM/ET/2013/MU/22	Afar	Megalle	Adahara	pos	neg	3	no

### *In vitro* adaptation in HMI-9 medium with horse serum

The highly concentrated trypanosome suspension in PSG was diluted to 2 x 10^5^ cells ml^−1^ in Hirumi’s modified Iscove’s medium 9 (HMI-9), complemented with 15% (v/v) heat-inactivated foetal bovine serum (Gibco, Belgium) and 5% (v/v) heat-inactivated horse serum (Gibco, Belgium) (abbreviated as HMI-9 (HS)) [40,41]. Parasites were seeded at 2 x 10^4^, 2 x 10^3^ and 2 x 10^2^ cells ml^−1^, in a total volume of 500 μl in a 48-well plate (Nunc, Denmark) and incubated at 37°C and 5% CO_2_. After 72 hours, a well, where trypanosome density had increased above 2 x 10^5^ cells ml^−1^, was used for further subpassage in 500 μl of HMI-9 (HS). The well with the highest density of viable parasites was then further maintained in HMI-9 without horse serum [40]. When possible, log phase growing *in vitro* cultures were scaled up in flasks (Nunc, Denmark) to obtain larger numbers of parasites for cryostabilisation, DNA extraction and *in vitro* drug sensitivity testing [42]. The *in vitro* growth curves of the different stocks were generated by seeding cells at 1 x 10^4^ cells ml^−1^ in 500 μl of HMI-9 in three replicate wells that were counted every 24 h. The doubling times (T_d_) were calculated from the exponential part of the curve using non-linear regression fitted with an exponential equation in GraphPad Prism 6 (GraphPad, version 6, USA).

### Molecular characterisation of parasite populations

DNA extraction of trypanosome sediments prepared from the *in vivo* expanded and the *in vitro* adapted populations was performed with DNA Isolation Kit (Roche Diagnostics, Germany) following the protocol recommended for isolation of DNA from mammalian tissue. From *T*.*b*. *brucei* AnTat 1.1^E^, *T*.*b*. *gambiense* LiTat 1.3, *T*.*b*. *gambiense* type II ABBA and *T*. *equiperdum* Dodola 940, DNA was extracted using the Maxwell 16 Tissue DNA Purification kit on a Maxwell 16 instrument according to the manufacturer's instructions (Promega, Belgium). DNA concentrations were measured using the Nanodrop ND-1000 UV-Vis spectrophotometer (NanoDrop Technologies, USA) and adjusted to 10 ng μl^-1^. A set of PCRs targeting VSG genes (RoTat 1.2 and JN 2118Hu), maxicircle genes (ND4, ND5, ND7 and A6), class A minicircles (miniA PCR) and class B minicircles (EVAB PCR) minisatellites (MORF-2REP), P2 adenosine transporter (AT1) and the F1-ATP synthase γ subunit were adopted to characterise the studied parasite populations [4,15,19,28,43–45]. Where applicable, the published PCR protocols were adjusted to the requirements of the HotStarTaq Plus DNA polymerase (Qiagen, Germany). Primer sequences, reaction mixture contents, cycling conditions and expected amplicon size are described and referenced in Table 2. All PCR amplifications were carried out in 200 μl thin-wall PCR tubes (ABgene, UK) in a T3 thermocycler 48 (Biometra, Germany). Ten μl of amplified products were electrophoresed in 1 to 2% agarose gel at 135 V for 30 min and afterwards stained with ethidium bromide for visualization under UV light. For direct sequencing, PCR was performed in 50–100 μl volumes and amplicons were cleaned up and concentrated using a PCR cleanup kit (QIAquick PCR Purification Kit, Qiagen, Germany) and sent out for bidirectional direct sequencing at the Genetic Sequencing Facility (VIB, Belgium) using the described PCR primers.

**Table 2:** PCRs used in the present study with target sequence, primer name and sequences, length of expected amplicon, reaction mixtures and cycling conditions. Reaction mixture 1: 25 μl containing 25 ng DNA, 1X CoralLoad buffer, 1.5 mM of MgCl_2_, 200 μM of dNTPs, 0.5 μM of each primer, 0.5 U of HotStar TaqPlus. Reaction mixture 2: 25 μl containing 25 ng DNA, 1X CoralLoad buffer, 1.5 mM of MgCl2, 200 μM of dNTPs, 1 μM of each primer, 0.5 U of HotStar TaqPlus. Reaction mixture 3: 25 μl containing 25 ng DNA, 1X CloneAmp HiFi PCR premix and 0.25 μM of each primer. bp: base pair, P: Plus DNA strand, M = Minus DNA strand.

Target sequence	Primers	Primer sequences	Amplicon length	Reaction mixture	Cycling conditions	Adapted from
VSG RoTat 1.2	ILO7957	5′-GCC ACC ACG GCG AAA GAC-3′	488 bp	1	95°C for 5 min and 35 cycles of 30 sec at 94°C, 30 sec at 58°C, 30 sec at 72°C and final extension for 5 min at 72°C	[43]
	ILO8091	5′-TAA TCA GTG TGG TGT GC-3′				[43]
VSG JN 2118Hu	Forward	5′-TTCTACCAACTGACGGAGCG-3′	273 bp	1	95°C for 5 min and 35 cycles of 30 sec at 94°C, 30 sec at 55°C, 30 sec at 72°C and final extension for 5 min at 72°C	[19]
	Reverse	5′-TAGCTCCGGATGCATCGGT-3′				[19]
Maxicircle A6	Forward	5′-AAAAATAAGTATTTTGATATTATTAAAG-3′	381 bp	2	95°C for 5 min and 30 cycles of 94°C for 1 min, 54°C for 1 min, and 72°C for 30 s followed by a final elongation step at 72°C for 8 min	[44]
	Reverse	5′-TATTATTAACTTATTTGATC-3′				[44]
Maxicircle ND4	Forward	5′-TGTGTGACTACCAGAGAT-3′	256 bp	2	Idem as above	[44]
	Reverse	5′-ATCCTATACCCGTGTGTA-3′				[44]
Maxicircle ND5	Forward	5′-TGGGTTTATATCAGGTTCATTTATG-3	400 bp	2	Idem as above	[28]
	Reverse	5′-CCCTAATAATCTCATCCGCAGTACG-3′				[28]
Maxicircle ND7	Forward	5′-ATGACTACATGATAAGTA-3	167 bp	2	Idem as above	[44]
	Reverse	5′-CGGAAGACATTGTTCTACAC-3′				[44]
Minicircle class A	MiniA	5′-GGGTTTTTTAGGTCCGAG-3′	1000 bp	1	95°C for 5 min and 35 cycles of 30 sec at 94°C, 30 sec at 58°C, 30 sec at 72°C and final extension for 5 min at 72°C	[15]
	Reverse MiniB	5′-CCGAAAATAGCACGTG-3’				[15]
Minicircle class B	EVAB1	5’-CACAGTCCGAGAGATAGAG-3’	436 bp	1	95°C for 5 min and 30 cycles of 30 sec at 94°C, 30 sec at 60°C, 60 sec at 72°C and final extension for 10 min at 72°C	[15]
	EVAB2	5’-CTGTACTCTACATCTACCTC-3’				[15]
Minisatellite MORF2-REP	P	5’TGCATGGCAATAGCGATGGGC-3’	repeated 102 bp sequence	1	95°C for 5 min and 30 cycles of denaturing at 94°C for 30 s, annealing at 60°C for 30 sec and extension at 72°C for 3 min. Elongation was continued for 72°C for 5 min	[4]
	M	5’ATCGTCACCTGGTGTACTTCTC-3’				[4]
TeAT1	TbAT1-F	5’-GAAATCCCCGTCTTTTCTCAC-3’	1600 bp	1	95°C for 5 min, 24 cycles of 1 min at 95°C followed by 1 min at 56°C and 2 min at 72°C, and final extension at 72°C for 5 min	[45]
	TbAT1-R-	5’-ATGTGCTGAGCCTTTTTCCTT-3’				[45]
F1-ATP synthase γ subunit	F1-ATP-F	5’-AACTGCAACGAAGCTTATGTCGGGCAAGCTTCGTC-3’	918 bp	3	98°C for 30 sand 35 cycles of 98°C for 15 s, 59.4°C for 15 s, 72°C for 20 s and 72°C for 5 min followed by cool down to 4°C	
	F1-ATP-R	5’-TAAATGGGCAGGATCCCTACTTGGTTACTGCCCCTTC-3’				

The full length sequence of the F1-ATP synthase γ subunit was cloned into a BamHI and HindIII double digested pHD309 vector using the In-Fusion Cloning kit (Clontech, Japan). Primers contained a F1-ATP synthase γ subunit specific sequence based on the *T*. *evansi* sequence of STIB 810 (EU185797) and a 5′ extension of 15 bp specific to the place of integration in pHD309, containing the restriction sites and sequence overlap with the vector, as required for the In-Fusion Cloning reaction. Proofreading-PCR was performed using the Clone-Amp HiFi PCR premix (Clontech, Japan). Amplicons were cleaned up (QIAquick PCR Purification Kit, Qiagen, Germany) before use in the In-Fusion protocol. The reaction products were transformed in Stellar competent cells according to the manufacturer's recommendations (Clontech, Japan). Transformant clones were checked for the presence of insert using colony PCR, cultured in LB medium, plasmid purified (QIAprep Spin Miniprep Kit, Qiagen, Germany) and at least 7 to 12 clones per transformation were bidirectionally sequenced at the Genetic Sequencing Facility (VIB, Belgium) using primers binding to pHD309.

### *In vitro* drug sensitivity testing

Melarsomine dihydrochloride (Cymelarsan, Sanofi Aventis, France) and isometamidium hydrochloride (Veridium, Ceva Santé Animale, Belgium) were prepared as 10 mg ml^−1^ stock solutions in distilled water. Dophanil powder (Dophanil, Docpharma, Belgium), containing 445 mg diminazene diaceturate and 555 mg antipyrine per gram, was concentrated to a 10 mg ml^−1^ diminazene diaceturate solution in DMSO (Sigma, Belgium). Suramin (Germanin, Bayer, Germany) was prepared as a 100 mg ml^−1^ in DMSO. A method to measure the IC_50_ values of compounds in 96-well plates was performed as described elsewhere [46]. Briefly, 2 × 10^4^ cells ml^−1^ from *in vitro* adapted stocks were exposed to seven threefold drug dilutions, ranging from 5000 to 7 ng ml^−1^ for suramin, 500 to 0.7 ng ml^−1^ for diminazene diaceturate and from 250 to 0.35 ng ml^−1^ for melarsomine dihydrochloride and isometamidium hydrochloride, in a total volume of 200 μl of HMI-9 medium. Next, the plate was incubated for 72 hours at 37°C with 5% CO_2_ followed by addition of 20 μl of resazurin (Sigma, Belgium; 12.5 mg in 100 ml PBS) for measuring trypanosomes viability. After a further 24 h incubation at 37°C and 5% CO_2_, fluorescence was measured (excitation λ = 560 nm; emission λ = 590 nm) with a VictorX3 multimodal plate reader using top reading (Perkin Elmer, Belgium) [42]. The results were expressed as the percent reduction in parasite viability compared to the parasite viability in control wells without drugs. The 50% inhibitory concentration (IC_50_) was calculated using non-linear regression fitted with a (log) inhibitor versus normalised response (variable slope) equation (GraphPad, version 6, USA). The IC_50_ values obtained from day 30 and day 60 *in vitro* cultures were compared using t-tests corrected for multiple testing according to the Holm-Sidak method (α = 0.05) (GraphPad, version 6, USA).

### Microscopic examination for presence of a kinetoplast in trypanosomes

Trypanosome populations at different stages of *in vivo* and *in vitro* expansion were examined for the presence of the kinetoplast using 4',6-diamidino-2-phenylindole (DAPI) staining. Briefly, live trypanosomes in PSG or *in vitro* culture medium were washed in PBS by centrifugation, deposited onto microscope slides, air dried and fixed with methanol for 30 min. Subsequently, the slides were rehydrated in PBS and mounted in 87% glycerol containing 1 μg ml^-1^ DAPI (Sigma, Belgium) [28]. Images were captured with an epifluorescence microscope (Olympus BX41, Olympus, Japan) equipped with a NU fluorescent cube (excitation: 360–370 nm and emission > 420 nm)) and Cell^˄^D software (Olympus, Japan). DAPI stained trypanosomes were grouped according to the number of kinetoplasts (K) and nuclei (N) present within each cell. The percentage of kinetoplastic cells in a DAPI stained slide was calculated on the basis of on average 300 examined trypanosomes, by dividing the sum of 1K1N + 2K1N + 2K2N cells by the sum of 1K1N + 2K1N + 2K2N + 0K1N + 0K2N cells. A two-tailed Spearman correlation matrix (using a confidence interval of 95%) was used to find the correlation between the percentage of kinetoplastic cells at day 30 and day 60 of *in vitro* culture and the respective IC_50_ value for a particular drug (GraphPad, version 6, USA).

### *In vivo* infectivity check

To check the *in vivo* infectivity of trypanosome populations that were cryostabilised after continuous propagation *in vitro* for 60 days, 5 x 10^6^ cells in 300 μl were inoculated in a single OF-1 mouse where after parasitaemia was checked as described above.

## Results

### Isolation of Ethiopian *T*. *evansi* stocks

Thirty cryopreserved buffy coat specimens from parasitologically positive dromedary camels were inoculated in immunosuppressed Swiss albino mice. In total, 22 parasite stocks originating from 22 different animals could be isolated and cryopreserved after 2 to 5 subpassages in mice. They were labelled as MCAM/ET/2013/MU/01 to MCAM/ET/2013/MU/22. Based on positivity in RoTat 1.2 PCR and EVAB PCR of the corresponding cryopreserved buffy coats, 20 of these stocks are *T*. *evansi* type A and 2 are *T*. *evansi* type B (Table 1) [20]. Copy cryovials of these primary isolates were brought to ITM, Antwerp and 14 were selected for further expansion in mice. The selection was based on their geographical origin and subtype: 12 type A stocks originated from different sampling stations in Afar and Tigray (MCAM/ET/2013/MU/01, 02, 04, 05, 06, 07, 08, 09, 11, 13, 15, 17) and two type B stocks (MCAM/ET/2013/MU/10 and 14) were from Awash Fentale in Afar. At peak parasitaemia, between 4 to 7 DPI, parasites were harvested, purified from blood using a mAECT column, washed with PSG and pelleted for DNA extraction and for *in vitro* culture adaptation.

### Molecular typing based on specific VSG sequences of *in vivo* expanded stocks

DNA extracts of *in vivo* expanded stocks were subjected to RoTat 1.2 PCR and JN 2118Hu PCR to identify the *T*. *evansi* type based on type-specific VSG sequences. In addition, the specificity of these PCRs was tested on DNA of other *Trypanozoon* strains (*T*.*b*. *brucei* AnTat 1.1^E^, *T*.*b*. *gambiense* LiTat 1.3, *T*.*b*. *gambiense* type II ABBA, *T*. *evansi* type A RoTat 1.2, *T*. *evansi* type B KETRI 2479 and *T*. *equiperdum* Dodola 940). Results are represented in Table 3. All the *in vivo* expanded stocks that originated from RoTat 1.2 PCR positive buffy coats, were also positive in RoTat 1.2 PCR (MCAM/ET/2013/MU/01, 02, 04, 05, 06, 07, 08, 09, 11, 13, 15 and 17). Direct sequencing of the 488 bp amplicons from these putative *T*. *evansi* type A stocks and the *T*. *evansi* RoTat 1.2 strain revealed 100% identity (in a 350 bp sequenced fragment) with the published RoTat 1.2 VSG sequence (AF317914), thus identifying them as *T*. *evansi* type A. Only one synonymous polymorphism (C699A) was found in MCAM/2013/ET/MU/04. The gel with the RoTat 1.2 PCR products from the purified trypanosomes showed a faint band of about 400 bp amplified in *T*. *evansi* KETRI 2479 and in MCAM/ET/2013/MU/10 and 14. Direct sequencing of these 400 bp amplicons failed. The PCR targeting the *T*. *evansi* type B specific VSG JN 2118Hu generated the expected amplicon in *T*. *evansi* type B KETRI 2479 and in MCAM/ET/2013/MU/10 and 14. Additionally, an amplicon was generated from MCAM/ET/2013/MU/15. Also for *T*.*b*. *brucei* AnTat 1.1^E^ and *T*.*b*. *gambiense* type II ABBA, amplicons of 273 bp were produced in the JN 2118Hu PCR. Direct sequencing of these amplicons revealed that the Ethiopian *T*. *evansi* type B MCAM/ET/2013/MU/10 and 14, *T*. *evansi* type B KETRI 2479 and *T*.*b*. *brucei* AnTat 1.1^E^ were 100% identical (in a 190 bp sequenced fragment) to the corresponding sequence of JN 2118Hu VSG (AJ870486). In *T*.*b*. *gambiense* type II ABBA, one synonymous mutation (G300A) was found.

**Table 3:** Genetic characteristics of the trypanosome populations studied. pos = positive, neg = negative, seq = sequence identity, n.a. = not applicable, n.d. = not done, (f) = faint, * amplification failed may be due to restricted elongation time in PCR protocol or probably high number of repeats present.

	RoTat 1.2		JN 2118Hu		Maxicircle PCR				Minicircle class		Fraction of kinetoplastic cells *in vivo*	Minisatellite profile
Trypanosome stock or strain	PCR	seq	PCR	seq	ND4	ND5	ND7	A6	A	B	%	MORF2-REP
*T*.*b*. *brucei* AnTat 1.1^E^	neg	n.a.	pos	identical	pos	pos	pos	pos	pos (f)	neg	n.d.	neg*.
*T*.*b*. *gambiense* LiTat 1.3	neg	n.a.	neg	n.a.	pos	pos	pos	pos	neg	neg	n.d.	7,11 (f)
*T*. *b*. *gambiense* ABBA	neg	n.a.	pos	G300A	pos	pos	pos	pos	neg	neg	n.d.	3
*T*. *evansi* RoTat 1.2	pos	identical	neg	n.a.	neg	neg	neg	neg	pos	neg	97	4,6
*T*. *evansi* KETRI 2479	neg	n.a.	pos	identical	neg	neg	neg	neg	neg	pos	98	3,5
*T*. *equiperdum* Dodola 940	neg	n.a.	neg	n.a.	pos	pos	pos	pos	neg	neg	n.d.	11(f)
MCAM/ET/2013/MU/001	pos	identical	neg	n.a.	neg	neg	neg	neg	pos	neg	97	7
MCAM/ET/2013/MU/002	pos	identical	neg	n.a.	neg	neg	neg	neg	pos	neg	98	6,7
MCAM/ET/2013/MU/004	pos	C699A	neg	n.a.	neg	neg	neg	neg	pos	neg	99	6,7
MCAM/ET/2013/MU/005	pos	identical	neg	n.a.	neg	neg	neg	neg	pos	neg	98	6,7
MCAM/ET/2013/MU/006	pos	identical	neg	n.a.	neg	neg	neg	neg	pos	neg	99	6,7
MCAM/ET/2013/MU/007	pos	identical	neg	n.a.	neg	neg	neg	neg	pos	neg	100	6,7
MCAM/ET/2013/MU/008	pos	identical	neg	n.a.	neg	neg	neg	neg	pos	neg	98	6,7
MCAM/ET/2013/MU/009	pos	identical	neg	n.a.	neg	neg	neg	neg	neg	neg	0%	6,7
MCAM/ET/2013/MU/010	neg	n.a.	pos	identical	neg	neg	neg	neg	neg	pos	98	3,4
MCAM/ET/2013/MU/011	pos	identical	neg	n.a.	neg	neg	neg	neg	pos	pos (f)	98	7
MCAM/ET/2013/MU/013	pos	identical	neg	n.a.	neg	neg	neg	neg	pos	neg	99	6,7
MCAM/ET/2013/MU/014	neg	n.a.	pos	identical	neg	neg	neg	neg	neg	pos	99	3,4
MCAM/ET/2013/MU/015	pos	n.d.	pos	n.d.	neg	neg	neg	neg	pos	pos	98	3,4,6,7
MCAM/ET/2013/MU/017	pos	identical	neg	n.a.	neg	neg	neg	neg	pos	pos (f)	99	6,7

### Morphological and genotypic kDNA status of the in vivo expanded stocks

Four PCRs that target maxicircle DNAs, of which three NADH-dehydrogenase subunits (ND4, ND5, ND7) and the ATPase subunit 6 (A6), and two PCRs that target class-specific minicircle sequences (miniA PCR and EVAB PCR) were run on DNA extracts of the purified trypanosomes (Table 3). All Ethiopian *T*. *evansi* stocks and *T*. *evansi* type A RoTat 1.2 and *T*. *evansi* type B KETRI 2479 were negative for all four maxicircle genes, while *T*.*b*. *brucei* AnTat 1.1^E^, *T*.*b*. *gambiense* LiTat 1.3, *T*.*b*. *gambiense* type II ABBA and *T*. *equiperdum* Dodola 940 were positive for all four maxicircle genes.

All stocks that contain RoTat 1.2 VSG, except MCAM/ET/2013/MU/09, were positive in miniA PCR. Additionally, weak amplification was seen in *T*.*b*. *brucei* AnTat 1.1^E^. MCAM/ET/2013/MU/10 and 14 were positive in EVAB PCR, confirming their identification as *T*. *evansi* type B as observed on their corresponding buffy coat specimens (Table 1). Additionally, EVAB PCR amplicons were detected in 3 stocks that were also positive for RoTat 1.2 VSG PCR suggesting a mixed infection with type A and B: a strong amplification was present in MCAM/ET/2013/MU/15, while a weak amplification was visible in MCAM/ET/2013/MU/11 and 17. The presence of kinetoplasts in the trypanosome cells was demonstrated using fluorescence microscopy with DAPI staining on *ex vivo* isolated trypanosomes (Table 3). *T*. *evansi* RoTat 1.2, *T*. *evansi* KETRI 2479 and all but one Ethiopian *T*. *evansi* stocks show a kinetoplast in > 96% of the cells. Stock MCAM/ET/2013/MU/09 was found to be akinetoplastic since only the nucleus of the trypanosomes was visible with DAPI.

### MORF2-REP minisatellite profile of the *in vivo* expanded stocks

**Fig 1:**
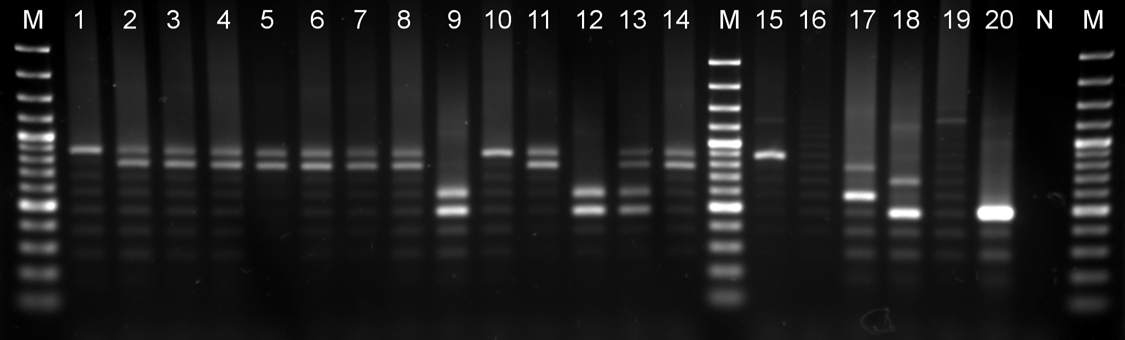
MORF2-REP profiles of Ethiopian *T*. *evansi* stocks and *T*. *evansi* and *T*. *brucei* reference strains. 1.5% agarose gel showing MORF2-REP minisatellite PCR amplicons. Lane M: 100 bp plus marker, lanes 1 to 14: Ethiopian *T*. *evansi* stocks MCAM/ET/2013/MU/01-02-04-05-06-07-08-09-10-11-13-14-15-17, lane 15: *T*.*b*. *gambiense* LiTat 1.3, lane 16: *T*.*b*. *brucei* AnTat 1.1^E^ lane 17: *T*. *evansi* type A (RoTat 1.2), lane 18: *T*. *evansi* type B (KETRI 2479), lane 19: *T*. *equiperdum* Dodola 940, lane 20: *T*. *b*. *gambiense* ABBA, lane N: negative control

In *T*. *evansi* RoTat 1.2, the MORF2-REP locus consists of 4 and 6 repeats, while in *T*. *evansi* KETRI 2479, 3 and 5 repeats were found (Table 3). *In vivo* expanded Ethiopian stocks of type A had either 1 allele (7 repeats) or 2 alleles (6 and 7 repeats), thus displaying a different pattern than *T*. *evansi* type A RoTat 1.2. The Ethiopian type B stocks MCAM/ET/2013/MU/10 and 14 contain 3 and 4 repeats, and thus have a pattern different from *T*. *evansi* type B KETRI 2479. MCAM/ET/2013/MU/15 showed a clear pattern of the Ethiopian type B (3 and 4 repeats), and double allele pattern of the Ethiopian type A (6 and 7 repeats). The other presumed mixed type A and type B stocks MCAM/ET/2013/MU/11 and 17 showed only the Ethiopian type A *T*. *evansi* pattern (Fig 1). DNA extracted from the buffy coats revealed similar MORF2-REP patterns as the *in vivo* expanded trypanosomes except for the buffy coat of MCAM/ET/2013/MU/15 that revealed only the Ethiopian type A MORF2-REP pattern. The other *Trypanozoon* strains showed the following patterns: *T*. *b*. *gambiense* LiTat 1.3 had 7 and 11 repeats, *T*.*b*. *gambiense* type II ABBA had 3 repeats, *T*. *equiperdum* Dodola 940 had 11 repeats, while no amplicons were generated from *T*.*b*. *brucei* AnTat 1.1^E^ under the giving PCR conditions.

### F1-ATP synthase γ subunit genotyping

Sequence analysis of in total 136 clones of the full length F1-ATP synthase γ subunit, amplified from DNA of the *in vivo* expanded Ethiopian stocks MCAM/ET/2013/MU/04, 06, 09, 10, 11, 13, 14, 15 and of *T*.*b*. *brucei* AnTat 1.1^E^, *T*.*b*. *gambiense* LiTat 1.3, *T*. *evansi* RoTat 1.2, *T*. *evansi* KETRI 2479, *T*. *b*. *gambiense* type II ABBA and *T*. *equiperdum* Dodola 940 revealed diverse homozygous and heterozygous nucleotide polymorphisms spread over the entire coding sequence (Table 4).

**Table 4:** F1-ATP synthase γ subunit single nucleotide polymorphism (SNP) observed within the studied trypanosome stocks and strains or retrieved from GenBank. Some SNPs were only present in *T*.*b*. *b*. TREU927 (G6A, C9T,C572G), T.b.b 29–13 (C149G, A168C, C866T) and T.b.b. STIB 920 (G738C) and are not represented in the table. del = deletion, GAN: GenBank accession number, * identical to all Ethiopian *T*. *evansi* type A stocks, ** identical to all Ethiopian *T*. *evansi* type B stocks. Blank spaces indicate no change and–indicates missing sequence information.

Stock/strain	A93G	C142T	C194T	A198G	G294A	T321C	A356T	T654C	T663C	G801T	T807C	G817C	841–843 GCT	A844T	T867G	A882G	T892C	GAN
*T*.*b*.*g*. DAL972	**G**			**G**						**T**						**G**		Tbg972.10.90
*T*.*b*.*g*. LiTat 1.3	**G**			**G**						**T**						**G**		KT934830
*T*.*b*.*g*. ABBA	**G**			**G**														KT934831
*T*. *ev*. RoTat 1.2*		**T**	**T**															KT934832
*T*. *ev*. RoTat 1.2*			**T**										**del**					KT934833
*T*. *ev*. STIB 810			**T**															EU185797
*T*. *ev*. STIB 810			**T**										**del**					EU185798
*T*. *ev*. KETRI 2479**						**C**					**C**				**G**			KT934834
*T*. *ev*. KETRI 2479**														**T**				KT934835
*T*. *ev*. KETRI 2479	-	-	-	-	-	-	-	-	-		**C**			**T**	**G**			EU185794
*T*. *eq* BoTat 1.1						**C**					**C**	**C**						EU185793
*T*. *eq*. STIB 841			**Y**		**R**	**C**	**W**	**Y**	**Y**						**G**		**C**	EU185792
*T*. *eq*. Dodola 940			**T**			**C**			**C**						**G**			KT934836
*T*.*b*.*b*. AnTat 1.1^E^			**T**			**C**			**C**						**G**			KT934837
*T*.*b*.*b*. AnTat 1.1^E^					**A**				**C**									KT934838
*T*.*b*.*b*. STIB 920						**C**			**C**		**C**				**G**			EU185791
*T*.*b*.*b*. 29–13	-		**T**			**C**			**C**						**G**		**C**	EU185790
*T*.*b*.*b*. TREU927	**G**				**A**	**C**	**T**	**C**										Tb927.10.180

The F1-ATP synthase γ subunit of *T*.*b*. *gambiense* LiTat 1.3 (KT934830) appeared homozygous and identical to the *T*.*b*. *gambiense* DAL972 sequence (Tbg972.10.90). *T*.*b*. *gambiense* type II ABBA (KT934831) appeared homozygous and differed in only 2 SNPs (G801T and A882G) from the *T*.*b*. *gambiense* sequence. *T*. *evansi* RoTat 1.2 and the Ethiopian stocks MCAM/ET/2013/MU/04, 06, 09,11 and 13 were heterozygous and revealed in one allele (KT934833), identical to the published full length *T*. *evansi* STIB 810 (EU185798) sequence, the deletion of nucleotides A841-843del. The second allele contained a C142T polymorphism (KT934832), that is not present in the wild-type *T*. *evansi* STIB 810 sequence (EU185797), but that could be identified in the genome sequence of the Chinese akinetoplastic *T*. *evansi* STIB 805 strain [9]. For *T*. *evansi* KETRI 2479 and the Ethiopian stocks MCAM/ET/2013/MU/10 and 14 we obtained heterozygous alleles, different from the partial sequence of *T*. *evansi* KETRI 2479 (EU185794). The first allele had the unique A844T polymorphism (KT934835), and differed from the second allele in 3 additional SNPs (T321C, T807C, T867G) that were also found in some *T*.*b*. *brucei* and *T*. *equiperdum*. Interestingly, the *in vivo* expanded stock of MCAM/ET/2013/MU/15 revealed alleles that belonged to *T*. *evansi* type A and type B. In contrast, when the original buffy coat of this stock was tested, only alleles of *T*. *evansi* type A were found. Finally, *T*. *equiperdum* Dodola 940 (KT934836) appeared homozygous and its single allele was identical to one of the two alleles found in *T*.*b*. *brucei* AnTat 1.1^E^ (KT934837), but differed in 5 SNPs with the sequence from *T*. *equiperdum* BoTat 1.1 (EU185793) and in 6 SNPs with *T*. *equiperdum* STIB 841 (EU185792). However, for the *T*. *equiperdum* STIB 841 strain, 5 of the 6 SNPs were ambiguous polymorphisms that do not rule out similarity to *T*. *equiperdum* Dodola 940.

### *In vitro* adaptation of Ethiopian *T*. *evansi* stocks

Fourteen Ethiopian *T*. *evansi* stocks, *T*. *evansi* RoTat 1.2 and *T*. *evansi* KETRI 2479 were expanded in mice and purified from blood at peak parasitaemia to initiate primary *in vitro* cultures in HMI-9 (HS) medium. After 96 hours, the initial 2x10^4^ cells ml^−1^ inoculum reached concentrations above 2x10^5^ cells ml^−1^ for all the different stocks. These cells were used for further *in vitro* propagation by subpassage in fresh medium. Over the next 72 hours, only MCAM/ET/2013/MU/09, 14 and 15, and *T*. *evansi* RoTat 1.2 and *T*. *evansi* KETRI 2479 showed proliferation. In contrast, slightly increased cell densities were observed for MCAM/ET/2013/MU/01, 04, 06 and 10. For all other strains not a single inoculum proliferated and longer incubation led to growth cessation.

Because the HMI-9 (HS) medium did not support sufficient *in vitro* culture growth for most of the Ethiopian *T*. *evansi* stocks, it was abandoned and replaced with HMI-9 without horse serum. *In vitro* adapted strains of *T*.*b*. *brucei* AnTat 1.1^E^ and *T*.*b*. *gambiense* LiTat 1.3 were cultured in HMI-9 in parallel. *In vitro* cultures were only considered adapted to HMI-9 medium when it was possible to maintain the parasites in continuous proliferation. To this extent, dense parasite cultures, containing 2–5 x 10^5^ cells ml^−1^, were subpassaged into new wells using serial fivefold dilutions in fresh medium. When these subpassages reached densities above 2 x 10^5^ cells ml^−1^ within a 48–96 hours period, the stock was considered adapted. The five stocks that already grew well in the HMI-9 (HS) medium continued proliferating when inoculated from the dense cultures at serial fivefold dilutions in HMI-9. These five stocks were considered to be *in vitro* adapted after 15 days of *in vitro* culture. Out of the four remaining stocks, only MCAM/ET/2013/MU/04 and 10 slowly regained the ability to proliferate in HMI-9 at a reduced subpassaging scheme using serial twofold dilutions. MCAM/ET/2013/MU/04 required 25 days to adapt, while MCAM/ET/2013/MU/10 was only fully adapted after day 35 of *in vitro* culture. Gradually increasing the culture volume allowed to obtain sufficient parasites from the adapted cultures for *in vitro* drug testing, DNA extraction, and cryostabilisation at day 30 (all, except MCAM/ET/2013/MU/10) and at day 60 of *in vitro* culture (all stocks).

**Fig 2:**
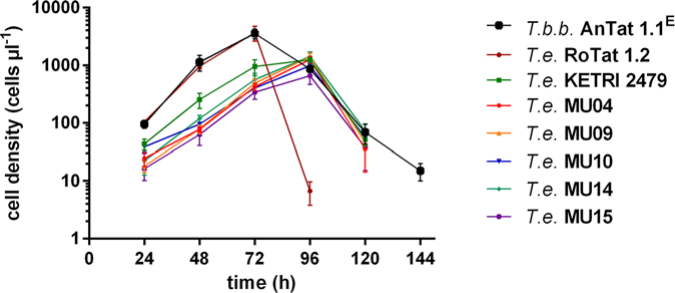
*In vitro* growth curve of trypanosome stocks and strains. *T*.*b*.*b*. = *T*.*b*. *brucei*, *T*.*e*. = *T*. *evansi*, MU = MCAM/ET/2013/MU.

DNA of the *in vitro* adapted stocks was subjected to RoTat 1.2 PCR, EVAB PCR and MORF2-REP PCR. All *in vitro* stocks had similar molecular profiles as their corresponding *in vivo* expanded parental stocks, except MCAM/ET/2013/MU/15. While the *in vivo* expanded stock of the latter was identified as a mixed infection of *T*. *evansi* type A and type B, the *in vitro* adapted stock (at day 30 and day 60 *in vitro* culture) was identified as pure *T*. *evansi* type B with the above mentioned PCRs and confirmed by cloning and sequencing of the F1-ATP synthase γ subunit. Thus, beside *T*. *evansi* RoTat 1.2 and *T*. *evansi* KETRI 2479, we achieved the *in vitro* adaptation of 2 Ethiopian type A stocks, 2 Ethiopian type B stocks and additionally ended up with a pure *T*. *evansi* type B *in vitro* adapted stock originating from a mixed type A and type B *in vivo* adapted stock. Growth curves were generated for *T*.*b*. *brucei* AnTat 1.1^E^ and all seven *in vitro* adapted stocks (Fig 2). *T*.*b*. *brucei* AnTat 1.1^E^ and *T*. *evansi* RoTat 1.2 had the shortest T_d_, 7.5 ± 0.3 h^-1^ and 7.7 ± 0.2 h^-1^ respectively, and reached the highest maximum population density (MPD) of ± 3–4 x 10^6^ cells ml^-1^, while *T*. *evansi* KETRI 2479 had a longer T_d_, 10.8 ± 0.2 h^-1^, and a lower MPD of ± 1 x 10^6^ cells ml^-1^. The Ethiopian type A stocks MCAM/ET/2013/MU04 and MU09 had a T_d_ of 11.2 ± 0.4 and 11.3 ± 0.4 respectively, and a MPD of ± 1 x 10^6^ cells ml^-1^. Similarly, the Ethiopian type B stocks MCAM/ET/2013/MU10, 14 and 15 had a T_d_ of 12.9 ± 0.5, 11.3 0.5 and 12.1 ± 0.6 respectively, and a MPD of ± 0.7–1 x 10^6^ cells ml^-1^ (Fig 2).

### *In vitro* drug sensitivity and relation to kDNA

**Fig 3:**
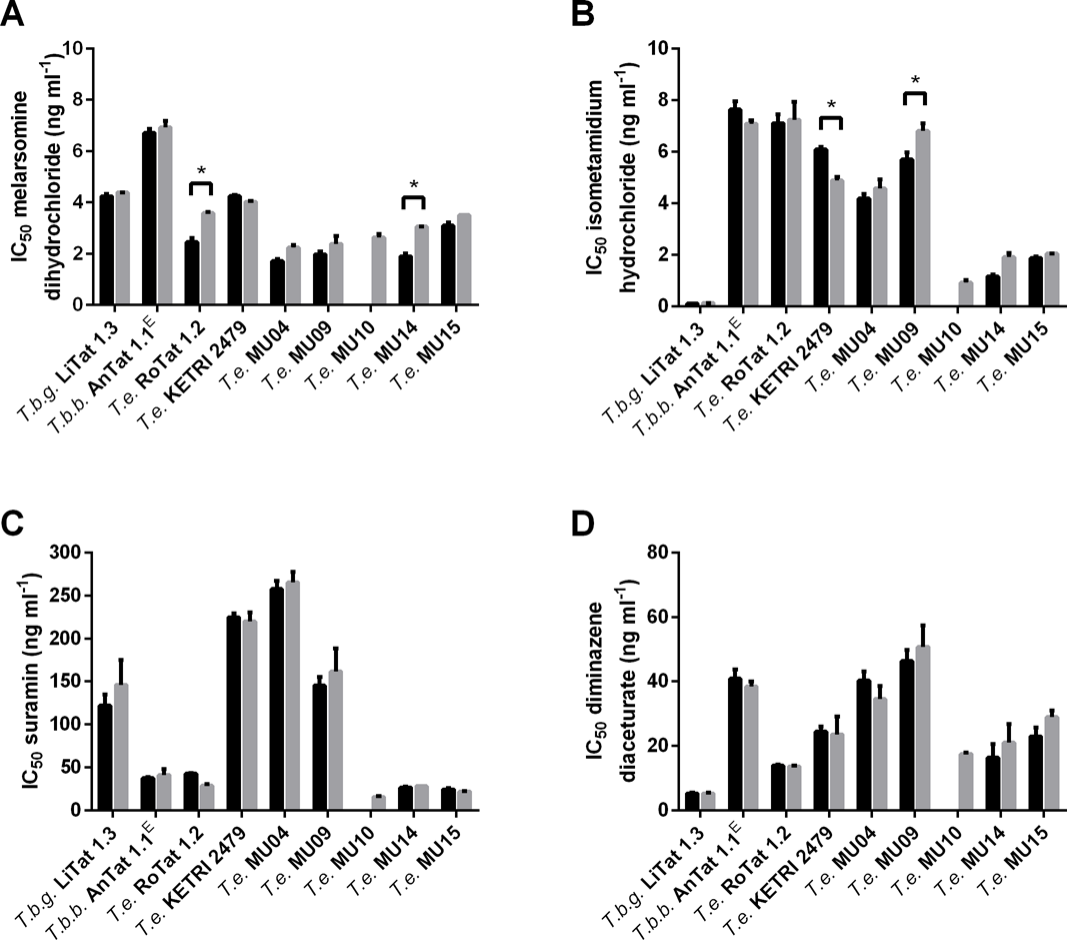
In vitro drug sensitivity of *T*. *evansi*. IC_50_ values for different drugs, with standard deviation error bars, obtained with different *Trypanosoma sp*. stocks and strains after 30 (black bars) and 60 days (grey bars) *in vitro* culture. Significant differences between IC_50_ values of 30 and 60 days *in vitro* cultures are indicated by an asterisk. *T*.*b*.*g*. = *T*.*b*. *gambiense*, *T*.*b*.*b*. = *T*.*b*. *brucei*, *T*.*e*. = *T*. *evansi*, MU = MCAM/ET/2013/MU. Panel A: IC_50_ values for melarsomine dihydrochloride. Panel B: IC_50_ values for isometamidium hydrochloride. Panel C. IC_50_ values for suramin and Panel D: IC_50_ values for diminazene diaceturate.

After day 30 and 60 of *in vitro* culture, IC_50_ values were determined for melarsomine dihydrochloride (Cymelarsan) (Fig 3A), isometamidium hydrochloride (Veridium) (Fig 3B), diminazene diaceturate (Dophanil) (Fig 3C) and suramin (Germanin) (Fig 3D). In general, non-significant differences (*p* > 0.05) were found between IC_50_ values recorded at day 30 and day 60 of *in vitro* culture, except for the melarsomine dihydrochloride IC_50_ values of *T*. *evansi* RoTat 1.2 and *T*. *evansi* MCAM/ET/2013/MU/14 and for the isometamidium hydrochloride IC_50_ values of *T*. *evansi* KETRI 2479 and *T*. *evansi* MCAM/ET/2013/MU/09 (*p* < 0.05). For comparison between the different stocks, the IC_50_ values of day 30 and day 60 of *in vitro* cultures were averaged. All Ethiopian *T*. *evansi* stocks had IC_50_ values for melarsomine dihydrochloride (IC_50_ 1.9–3.3 ng ml^-1^) that were similar to those of *T*.*b*. *gambiense* LiTat 1.3 (IC_50_ 4.3 ng ml^-1^), *T*.*b*. *brucei* AnTat 1.1^E^ (IC_50_ 6.8 ng ml^-1^), *T*. *evansi* RoTat 1.2 (IC_50_ 3.0 ng ml^-1^) and *T*. *evansi* KETRI 2479 (IC_50_ 4.1 ng ml^-1^). For isometamidium hydrochloride, the IC_50_ values of the Ethiopian *T*. *evansi* (IC_50_ 0.6–6.2 ng ml^-1^) fall within the range of *T*.*b*. *gambiense* LiTat 1.3 (IC_50_ 0.1 ng ml^-1^), *T*.*b*. *brucei* AnTat 1.1^E^ (IC_50_ 7.3 ng ml^-1^), *T*. *evansi* RoTat 1.2 (IC_50_ 7.1 ng ml^-1^) and *T*. *evansi* KETRI 2479 (IC_50_ 5.5 ng ml^-1^). However, the two Ethiopian *T*. *evansi* type A stocks (IC_50_ 4.3–6.2 ng ml^-1^) appear to be threefold less sensitive that the three type B stocks (IC_50_ 0.6–1.9 ng ml^-1^). For suramin, large differences in IC_50_ values were found among the Ethiopian *T*. *evansi* (IC_50_ 15.9–261.5 ng ml^-1^) stocks and among the other strains: *T*.*b*. *brucei* AnTat 1.1^E^ (IC_50_ 39.5 ng ml^-1^) and *T*. *evansi* RoTat 1.2 (IC_50_ 35.8 ng ml^-1^) appear highly susceptible, while *T*.*b*. *gambiense* LiTat 1.3 (IC_50_ 134.0 ng ml^-1^) and *T*. *evansi* KETRI 2479 (IC_50_ 222.4 ng ml^-1^) are less susceptible. The two Ethiopian *T*. *evansi* type A (IC_50_ 153.5–261.5 ng ml^-1^) appear to be tenfold less sensitive than the three type B (IC_50_ 15.9–27.6 ng ml^-1^). For diminazene diaceturate, the IC_50_ values of all Ethiopian *T*. *evansi* (IC_50_ 17.5–48.5 ng ml^-1^) are higher than those of *T*.*b*. *gambiense* LiTat 1.3 (IC_50_ 5.2 ng ml^-1^) and *T*. *evansi* RoTat 1.2 (IC_50_ 13.8 ng ml^-1^), but similar to *T*.*b*. *brucei* AnTat 1.1^E^ (IC_50_ 39.6 ng ml^-1^) and *T*. *evansi* KETRI 2479 (IC_50_ 24.0 ng ml^-1^). The two Ethiopian *T*. *evansi* type A (IC_50_ 37.4–48.5 ng ml^-1^) appear to be twofold less sensitive than the three type B (IC_50_ 17.5–25.9 ng ml^-1^). Direct sequencing of the full length TeAT1 PCR amplicons of MCAM/ET/2013/MU/04, 09, 10, 14, and 15, *T*. *evansi* type A RoTat 1.2 and *T*. *evansi* Type B KETRI 2479 revealed no polmorphisms to the wild-type *TeAT1* sequence (AB124588).

**Fig 4:**
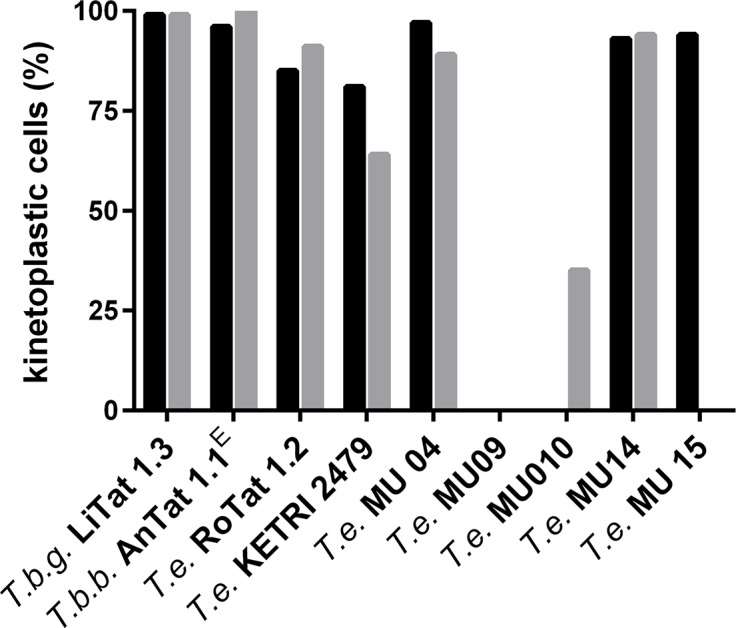
Percentage of kinetoplastic cells within *T*. *evansi* populations. Percentage of kinetoplastic cells visualised after DAPI staining and fluorescence microscopy within populations after 30 (black bars) and 60 days (grey bars) *in vitro* propagation. *T*.*b*.*g*. = *T*.*b*. *gambiense*, *T*.*b*.*b*. = *T*.*b*. *brucei*, *T*.*e* = *T*. *evansi*, MU = MCAM/ET/2013/MU.

DAPI staining was performed on *in vivo* and *in vitro* propagated stocks (Fig 4). *In vitro* culture did not change the percentage of kinetoplastic cells in *T*.*b*. *gambiense* LiTat 1.3 (99%), *T*.*b*. *brucei* AnTat 1.1^E^ (99%) and MCAM/ET/2013/MU/09 (0%). On the other hand, already after 30 days *in vitro* culture a decrease in the percentage of kinetoplastic cells was observed in *T*. *evansi* RoTat 1.2 (89%), *T*. *evansi* KETRI 2479 (81%), MCAM/ET/2013/MU/04 (97%), 14 (93%) and 15 (94%) compared to non-*in vitro* adapted trypanosomes. After 60 days of *in vitro* culture, the percentage of kinetoplastic cells dropped even further for *T*. *evansi* KETRI 2479 (64%), MCAM/ET/2013/MU/04 (89%) and 10 (35%). No significant correlation was observed between the percentage of kinetoplastid cells of all *in vitro* adapted *T*. *evansi* stocks (including day 30 and day 60) and their IC_50_ values for melarsomine dihydrochloride (ρ = -0.13, *p* = 0. 67), isometamidium hydrochloride (ρ = -0.324, *p* = 0.278), suramin (ρ = -0.097, *p* = 0.752) and diminazene diacetureate (ρ = -0.355, *p* = 0.233). These data suggest that among the *in vitro* adapted Ethiopian *T*. *evansi* stocks there is no relation between the drug sensitivity and the presence of kinetoplast DNA. Furthermore, their loss of kDNA does not seem to influence rodent infectivity since all cryostabilates made from day 60 *in vitro* cultures remained infective for mice with detectable parasitaemia at 4–5 DPI.

## Discussion

Previous molecular and serological studies revealed that trypanosome infections in camels from Northern Ethiopia are caused by either RoTat 1.2 PCR or EVAB PCR positive parasites. In some instances amplicons of both PCRs were detected within the same buffy coat extract, suggesting the occurrence of mixed infections [20]. The present study was undertaken to isolate the trypanosomes from camels carrying apparent single infections through inoculation of their buffy coats in immunosuppressed mice. The *in vivo* inoculation led to the successful isolation of 22 stocks, out of which 14 were selected on the basis of their geographical origins for further investigations (5 stocks from Tigray and 9 stocks from Afar). Next, we performed an in-depth comparative molecular analysis on DNA extracts from the isolated parasite stocks using diverse PCRs. Furthermore, we analysed the specificity of each of these PCRs on a collection of *Trypanozoon* strains.

The RoTat 1.2 VSG sequence can be used to characterise *T*. *evansi* type A [25,43]. In our collection, all buffy coats positive in RoTat 1.2 PCR yielded *in vivo* isolated stocks that were RoTat 1.2 PCR positive but that were negative in the maxicircle gene targeting PCRs. Furthermore, with the exception of the akinetoplastic stock MCAM/ET/2013/MU/09, all these strains had type A minicircles. MCAM/ET/2013/MU/09 may be naturally akinetoplastic since the DNA extracted from the original buffy coat was negative in all PCRs targeting kinetoplast DNA. The occurrence of naturally akinetoplastic strains was previously documented in Latin America and China [12–14,47]. One stock (MCAM/ET/2013/MU/04) contained a SNP in its RoTat 1.2 VSG PCR amplicon. SNPs in RoTat 1.2 amplicons were previously reported in Egypt but do not necessarily lead to a negative result in RoTat 1.2 based antibody detection tests. This was also the case for the camel from which MCAM/ET/2013/MU/04 was isolated [48,49].

Initially defined by minicircle class B, identification of *T*. *evansi* type B is possible with EVAB PCR that amplifies a fragment of this minicircle [15]. Additionally, it was proposed that the VSG JN 2118Hu, first described in a Kenyan *T*. *evansi* strain, is a specific marker for *T*. *evansi* type B [19].

In our collection, 2 buffy coat extracts that were positive in EVAB PCR yielded *in vivo* isolated stocks that were EVAB PCR positive as well. Interestingly, an EVAB PCR amplicon was also detected in three additional *in vivo* expanded stocks that were RoTat 1.2 PCR positive but for which the corresponding buffy coats were EVAB PCR negative. These three stocks might be mixed infections. JN 2118Hu VSG PCR appeared to be less sensitive because it detected only 3 out of 5 EVAB PCR positive isolated stocks. Furthermore, the JN 2118Hu VSG PCR appeared to be less specific since *T*.*b*. *brucei* AnTat 1.1^E^ and *T*.*b*. *gambiense* type II ABBA were also positive in this PCR. None of the EVAB PCR positive isolated stocks contained maxicircle DNA and they were all negative in miniA PCR, except for the three mixed infections. Therefore, we conclude that we isolated at least two “pure” *T*. *evansi* type B stocks from Ethiopian camels, decades after the initial isolation of *T*. *evansi* type B from camels in Kenya [15].

We used the minisatellite locus MORF2-REP to verify whether both putative mixed stocks, that were positive in RoTat 1.2 PCR and EVAB PCR, were real mixed infections or hybrids between *T*. *evansi* type A and B. The Ethiopian isolates clustered in two classes of *T*. *evansi* type A, of which one with a previously described heterozygous profile (6 and 7 repeats) and one with a homozygous profile (7 repeats). The Ethiopian *T*. *evansi* type B stocks had a heterozygous profile (3 and 4 repeats) differing from the only known profile described for Kenyan type B isolates (3 and 5 repeats) [50]. In one of the mixed infections we observed a profile that can be interpreted as a mixture of Ethiopian type A and type B, while the others only revealed the Ethiopian type A pattern. These results prove that we are dealing with mixed infections and not with hybrids between *T*. *evansi* type A and type B. To exclude that these apparent mixed infections represent cross-contamination with genetic material, we attempted *in vitro* cultivation of the *in vivo* expanded stocks.

Previously we have shown that addition of 1,1% methylcellulose to HMI-9 greatly helps the *in vitro* adaptation of *Trypanozoon* strains, including *T*.*b*. *gambiense* and *T*. *evansi* RoTat 1.2 [40]. However, to avoid the use of this highly viscous medium we preferred the use of horse serum to adapt *T*. *evansi* stocks as is suggested in previous reports [51–53]. While this approach proved to be successful for all type B stocks, only two out of nine Ethiopian *T*. *evansi* type A could be adapted. Interestingly, in the case of mixed stock MCAM/ET/2013/MU/15, this medium selected *T*. *evansi* type B out of the mixed population. While only the type A infection was detected in the buffy coat DNA extract, both types could be detected in the *in vivo* expanded stock DNA, but eventually only type B was detected in the *in vitro* adapted stock.

Gillingwater and colleagues reported on the drug sensitivity profiles of a panel of *T*. *evansi* and *T*. *equiperdum* strains where they considered *T*. *evansi* STIB 806K to be a reference sensitive strain for suramin (IC_50_ 70.4 ng ml^-1^), diminazene diaceturate (IC_50_ 4.5 ng ml^-1^) and melarsomine dihydrochloride (IC_50_ 1.4 ng ml^-1^). They reported drug resistance in two *T*. *evansi* stocks with an IC_50_ for suramin > 10000 ng ml^-1^ (STIB 780 and STIB 781), and in the *T*. *equiperdum* OVI strain, with an IC_50_ for diminazene diaceturate of 302 ng ml^-1^ and an IC_50_ for melarsomine dihydrochloride of 17.6 ng ml^-1^ [46]. The only strain that is shared between their panel and our collection is *T*. *evansi* RoTat 1.2, which despite different approaches in the experimental testing, yielded corresponding IC_50_ values, especially for diminazene diaceturate and melarsomine dihydrochloride, thus facilitating comparison between both studies. In our Ethiopian *T*. *evansi* collection, no resistance against melarsomine dihydrochloride was found. However, some stocks appeared to have raised IC_50_ values for suramin (> 200 ng ml^-1^) and diminazene diaceturate (> 50 ng ml^-1^). The IC_50_ values that we observe for *T*.*b*. *gambiense* LiTat 1.3 and the Ethiopian *T*. *evansi* type B are similar to the *in vitro* IC_50_ value of 0.82 ng ml^-1^ found by Sahin and coworkers for *T*. *congolense* IL3000 which is sensitive to isometamidium (Veridium) *in vivo* [54]. In the same study, an *in vitro* IC_50_ of 11.06 ng ml^-1^ is reported for *T*.*b*. *brucei* AnTat 1.1 strain, which is slightly higher than the value that we obtained in experiments with our *T*.*b*. *brucei* AnTat 1.1 strain and the other *T*. *evansi* stocks [54]. Nevertheless, defining our *T*. *evansi* stocks as either sensitive or resistant based solely on the *in vitro* drug sensitivity results may be too audacious, given the fact that IC_50_ values were determined in only one assay, the resazurin viability assay [55–57]. Therefore, an *in vivo* drug sensitivity profile of all our *Trypanozoon* strains against the commonly used trypanocides remains to be elucidated. Interestingly, both Ethiopian *T*. *evansi* type A stocks appear to be less susceptible to suramin, diminazene diaceturate and isometamidium hydrochloride than the three type B stocks. In *T*.*b*. *brucei*, resistance against suramin and isometamidum hydrochloride has been linked to several proteins [58,59], while resistance to diamidine and melaminophenyl classes of drugs is attributed to the transporter protein TbAT1 and the aquaporin AQP2 [60–62]. The lower sensitivity to diminazene diaceturate was not caused by mutations in the *T*. *evansi TeAT1* [63].

Interestingly, DAPI staining of the trypanosomes indicated slight to severe loss of the kDNA in all *in vitro* adapted *T*. *evansi* stocks, when compared to *in vivo* adapted stocks. The loss of kDNA in *in vitro* cultured *T*. *evansi* is a phenomenon that has been known for a long time [10,15,55,64]. Non-vital loss of the kinetoplast is made possible by mutations in the F1-ATP synthase γ subunit of *T*. *evansi* allowing to uncouple from the Fo subunit and effectively circumventing the requirement for mitochondrial gene expression [65]. Furthermore, it has been shown that the expression of certain *T*. *evansi* F1-ATP synthase γ subunit coding sequences in *T*. *brucei* allows this species to survive loss of its kDNA after chemical treatment [28]. Moreover, in such genetically modified *T*. *brucei*, independence of kDNA maintenance and expression is associated with multidrug resistance [30]. In our collection of *T*. *evansi* stocks we did not observe differences in drug sensitivity between populations that were partially or completely akinetoplast confirming earlier evidence that the presence or absence of kDNA is irrelevant within this context [30,55].

Recently, Carnes *et al*. showed that SNPs in the F1- ATP γ subunit could be used to genotypically support the multiple origins of at least 4 dyskinetoplastic *T*. *evansi/T*. *equiperdum* lineages: one major group of RoTat 1.2 VSG positive *T*. *evansi/T*. *equiperdum* type A, and three very small groups each represented by only a single strain: *T*. *evansi* type B KETRI 2479, *T*. *equiperdum* BoTat and *T*. *equiperdum* OVI [9]. All Ethiopian *T*. *evansi* type A had the corresponding mutation of the type A group. The Ethiopian type B *T*. *evansi* shared a similar profile as KETRI 2479. Finally, the Ethiopian *T*. *equiperdum* strain Dodola, which had some maxicircle genes but was negative for both type A and type B markers revealed an F1-ATP synthase sequence similar to *T*.*b*. *brucei* AnTat 1.1^E^ strain, thus likely belongs to the same dyskinetoplastic group as *T*. *equiperdum* OVI [9,28].

In conclusion, our study shows that the apparent *T*. *evansi* type that is detected in a buffy coat of an infected camel does not necessarily represent the full diversity that is present in the infected animal. Moreover, the fact that 5 out of 22 new *T*. *evansi* isolates from camel in Ethiopia contain *T*. *evansi* type B may be an indication that is more widespread than currently known. The inoculation of the trypanosomes in immunosuppressed mice may allow the propagation of mixed populations. In contrast, *in vitro* cultivation seems to reduce the diversity by selecting for only one particular type, in our study *T*. *evansi* type B. Secondly, our study addresses some drawbacks of current molecular markers for *T*. *evansi* genotyping. To rely solely on VSG markers or kDNA markers for the molecular identification of *T*. *evansi* may be misleading due to possible recombinations occurring in VSG genes and to the presence of akinetoplastic *T*. *evansi* stocks. In this regard, we confirm that the F1-ATP synthase γ subunit gene, that is not related to the VSG repertoire nor to the presence of kDNA, may become an interesting target for genotyping *T*. *evansi* stocks in areas where both types overlap and where mixed infections can occur. Nevertheless, it is not possible to separate the Ethiopian *T*. *equiperdum* from *T*. *brucei* on the basis of this target gene. Thirdly, no evidence of *in vitro* drug resistance was found in our collection of *T*. *evansi* type A and type B stocks. The presence or partial absence of kDNA in the *in vitro* adapted *T*. *evansi* stocks did not correspond with the drug sensitivity phenotype.
